# Spatial heterogeneity of intracardiac 4D relative pressure fields during diastole

**DOI:** 10.1186/1532-429X-15-S1-M13

**Published:** 2013-01-30

**Authors:** Jonatan Eriksson, Ann Bolger, Tino Ebbers, Carl Johan Carlhall

**Affiliations:** 1 Department of Medical and Health Sciences, Linköping University, Linköping, Sweden; 2 Center for Medical Image Science and Visualization (CMIV), Linköping University, Linköping, Sweden; 3 Department of Clinical Physiology UHL, County Council of Östergötland, Linköping, Sweden; 4 Department of Medicine, University of California, San Francisco, CA, USA

## Background

Blood flow within the cardiovascular system is driven by pressure differences, where blood accelerates from higher to lower pressure areas. Invasive methods of pressure measurement, which are commonly applied to assessment of diastolic function, may not capture the heterogeneity of regional intracardiac pressure differences. We utilized pressure fields based on time-resolved 3D CMR data to investigate the timing and distribution of intracardiac pressure gradients in the left heart throughout diastole.

## Methods

12 healthy subjects (5 female, age 47±17 y.o. (mean±sd)) underwent MRI examination (1.5T, Philips Achieva) where 4D velocity and morphological short (SA) and long axis bSSFP data were acquired. Acquisition parameters for the velocity sequence were: TE 3.7 ms, TR 6.3 ms, spatial resolution 3x3x3 mm3, and k-space segmentation factor 2. This resulted in a temporal resolution of 50.4 ms. The field-of-view was adjusted to fit each subject.

**Figure 1:**
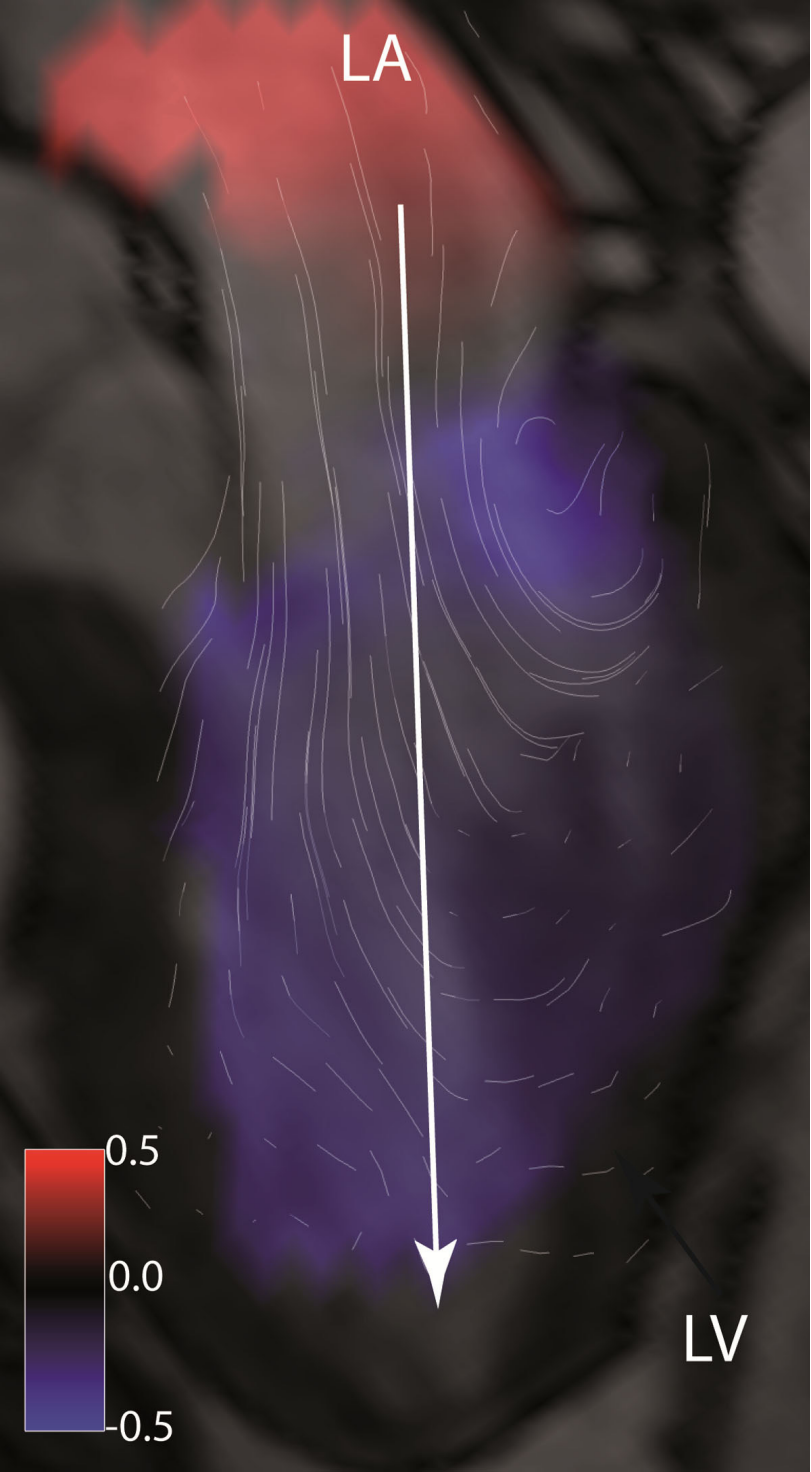
Visualization of relative pressure (mmHg) at TimeOfMaxPressure in a healthy 61 y.o. male with a heart rate of 61 bpm. A morphological three-chamber image is included for orientation. Certain data were extracted from a line through the 3D data set from left atrium (LA) to left ventricular (LV) apex (arrow).

The left ventricle (LV) and atrium (LA) were segmented at all diastolic time frames using freely available software Segment (http://segment.heiberg.se). The segmentation was used as boundary condition for computation of the 4D relative pressure field, based on the pressure Poisson equation (Ebbers and Farnebäck, 2009 JMRI). The pressure data were visualized using the commercial software package EnSight (figure 1). The pressure range for the entire LA and LV volume at each diastolic time frame was calculated and the maximum value identified in each subject. Further, the pressure range in the full segmented LV volume was calculated and related to the pressure range along a line from LA to LV (figure 1), at the time of maximum positive pressure gradient between LA and LV during early filling (TimeOfMaxPressure).

## Results

**Figure 2:**
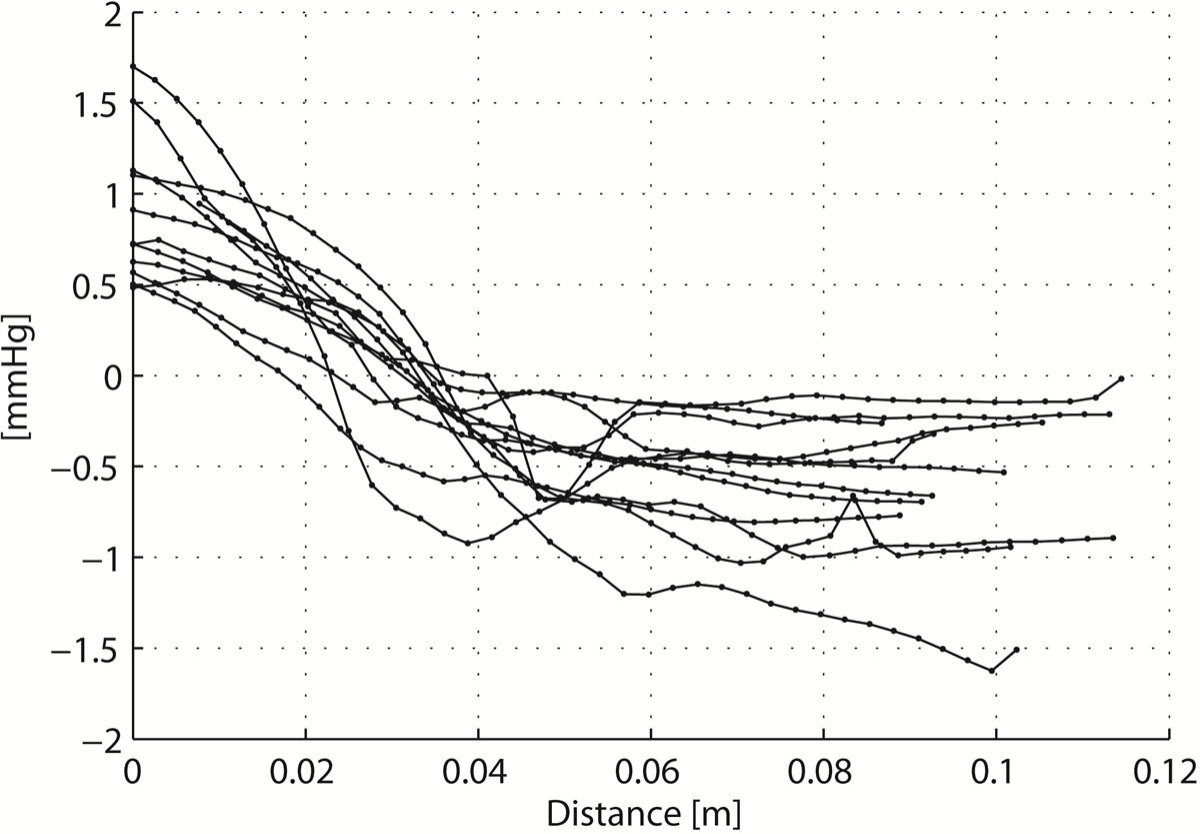
Relative pressure (mmHg) along a line between left atrium (LA) and left ventricular (LV) apex, (figure 1) at TimeOfMaxPressure. Data represent 12 healthy subjects. The x-axis show distance in meters from the start of the line in the atrium to the ventricular apex.

The maximum pressure range anywhere in the combined LA and LV volume at any time during diastole was 3.2±1.1 mmHg. At TimeOfMaxPressure, the pressure range anywhere in the LV volume was 2.0±1.0 mmHg. At the same time point, the pressure range along the atrium-to-apex line was 1.7±0.6 mmHg (figure 2), which represented 89±21% of the pressure range anywhere in the LV volume.

## Conclusions

This CMR study implies that intracardiac pressure gradients are spatially heterogenous during the diastolic phase. These results emphasize the benefit of pressure assessment from methods that take into account the 3D nature of the intracardiac pressure field. These findings may also impact the interpretation of clinical catheter-based intracardiac pressure measurements.

## Funding

This study was funded by the Swedish heart-lung foundation and the Swedish research council.

